# Preparation, Characterization and Antibacterial Property Analysis of Cellulose Nanocrystals (CNC) and Chitosan Nanoparticles Fine-Tuned Starch Film

**DOI:** 10.3390/molecules27238542

**Published:** 2022-12-04

**Authors:** Zilong Deng, Zixuan Wu, Xiao Tan, Fangkun Deng, Yaobang Chen, Yanping Chen, Hongcai Zhang

**Affiliations:** 1 State Key Laboratory for Pollution Control, School of Environmental Science and Engineering, Tongji University, Shanghai 200092, China; 2 College of Food Science and Technology, Shanghai Ocean University, Shanghai 201306, China; 3 Jiangxi New Dragon Biotechnology Co., Ltd., Yichun 336000, China; 4 Sibang Environmental Protection Technology Co., Ltd., Yichun 336000, China; 5 School of Agriculture and Biology, Shanghai Jiao Tong University, Shanghai 200240, China

**Keywords:** cellulose nanocrystals, chitosan nanoparticles, nanocomposite film, antibacterial properties

## Abstract

To improve the mechanical and antibacterial properties of traditional starch-based film, herein, cellulose nanocrystals (CNCs) and chitosan nanoparticles (CS NPs) were introduced to potato starch (PS, film-forming matrix) for the preparation of nanocomposite film without incorporation of additional antibacterial agents. CNCs with varied concentrations were added to PS and CS NPs composite system to evaluate the optimal film performance. The results showed that tensile strength (TS) of nanocomposite film with 0, 0.01, 0.05, and 0.1% (*w*/*w*) CNCs incorporation were 41, 46, 47 and 41 MPa, respectively. The elongation at break (EAB) reached 12.5, 10.2, 7.1 and 13.3%, respectively. Due to the reinforcing effect of CNCs, surface morphology and structural properties of nanocomposite film were altered. TGA analysis confirmed the existence of hydrogen bondings and electrostatic attractions between components in the film-forming matrix. The prepared nanocomposite films showed good antibacterial properties against both *E. coli* and *S. aureus*. The nanocomposite film, consist of three most abundant biodegradable polymers, could potentially serve as antibacterial packaging films with strong mechanical properties for food and allied industries.

## 1. Introduction

Biodegradable packaging material has attracted considerable attention due to its reduction of non-degradable waste and environmental sustainability [1]. The abundant biopolymers, including starch [2,3], gum [4], soy protein isolate [5], alginate [6,7], gelatin [8,9] and polyvinyl alcohol (PVA) [10,11], have been widely applied as bio-based packaging materials. However, these biopolymers (e.g., potato starch, PS) exhibit characteristic defects including high hydrophilicity, poor moisture barrier, low mechanical strength and lack of antibacterial properties [2,3,12].

Incorporation of antimicrobial nanocomposites can extend the shelf life of products by controlling the undesirable growth of foodborne pathogens [13]. Previous studies reported the strong antibacterial activity of chitosan (CS) nanoparticles (NPs), derived from fabrication of positively charged CS and negatively charged sodium triphosphate (TPP) through electrostatic interactions [14,15]. The CS NPs with nano-scaled diameter and the large surface area attach to the bacterial surface and contribute to the death of cells, resulting in antibacterial activity [16,17,18,19]. However, CS NPs are prone to aggregate and suffer uncontrollable release, leading to poor stability and limited bactericidal activity [16].

As the most abundant bio-based polysaccharide on Earth, cellulose and its nanoscale derivatives are ideal reinforcing polymer for food preservation. Among the nanocellulose family fabricated from acid hydrolysis for minimizing the amorphous region, CNCs consist of unique properties including low density, high stiffness, aspect ratio and surface area with biocompatibility and biodegradability in the absence of harmfulness to environment [20]. Preparation, characterization and application of CNCs as either mechanical enhancers or fillers were mainly considered for the enhancement of biodegradable polymers (e.g., chitosan [21], starch [22], sodium alginate [23] and pectin [24]) by fine-tuning the concentration and surface characteristic in previously studies. Moreover, CNCs could also apply as a typical stabilizer to improve the stability and antibacterial activity of lysozyme [25], oleic acid [26] and trace metallic nanoparticles [27].

In this work, we hypothesized that the fine-tuned concentration of CNCs could prevent the aggregation of CS NPs (antibacterial agent) through stabilization in PS matrix. To the best of our knowledge, this is the first-ever report to combine both CNCs and CS NPs for the preparation of potato starch-based film. The objectives of the study are to (1) characterize the surface morphology and structural properties and (2) investigate physicochemical and antibacterial properties of CNCs and CS NPs incorporated PS composite films with proposed mechanisms. It is expected that developed nanocomposite film may potentially be applied for food preservation and biodegradable packaging films.

## 2. Results and Discussions

### 2.1. Physicochemical Properties of CNCs Nanocomposite Film

**Figure 1:**
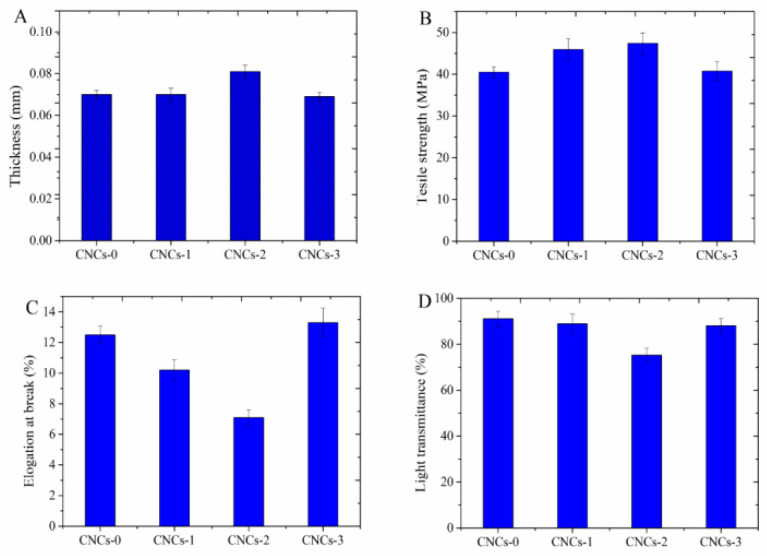
Thickness (**A**), tensile strength (**B**), elongation at break (**C**) and light transmittance (**D**) of CNCs nanocomposite film. a, b, c, and d represented the nanocomposite film with 0, 0.01, 0.05 and 0.1% (*w*/*w*) of CNCs incorporation, respectively.

Thickness, TS, EAB and light transmittance of CNCs nanocomposite film are presented in Figure 1. Thickness of CNCs-0, CNCs-1, CNCs-2 and CNCs-3 were 0.069, 0.07, 0.081 and 0.069 mm, respectively. Film thickness was elevated with increasing CNCs concentration but reduced at the concentration of 0.1%. This was probably because of significant amount of CNC incorporation to polymeric matrix. TS of CNCs-0, CNCs-1, CNCs-2 and CNCs-3 were 40.50, 45.94, 47.41 and 40.77 MPa, respectively. EAB of CNCs nanocomposite film were calculated to be 12.5, 10.2, 7.1 and 13.3%, respectively. The illustrated trends were consistent for film thickness and TS, in contrast to EAB measurements. The low TS of CNCs-3 might be due to the aggregation of CNC, leading to reduction of free volume and molecular mobility of the polymer [28,29]. Similar trend was reported by Balakrishnan et al. that the reinforcing properties of nanocomposite film was improved by the inclusion of CNCs to thermoplastic starch [30]. González et al. also reported that stiffness of starch-based nanocomposite hydrogels was enhanced due to the addition of CNCs (2.5 and 5 wt%) [31]. Light transmittance of CNCs nanocomposites were 91.13, 88.92, 75.29, and 88.11%, respectively. The lowest value of CNCs-2 was probably because of optimal incorporation concentration of CNCs as filler to enhance the crystallinity of composite film.

**Figure 2:**
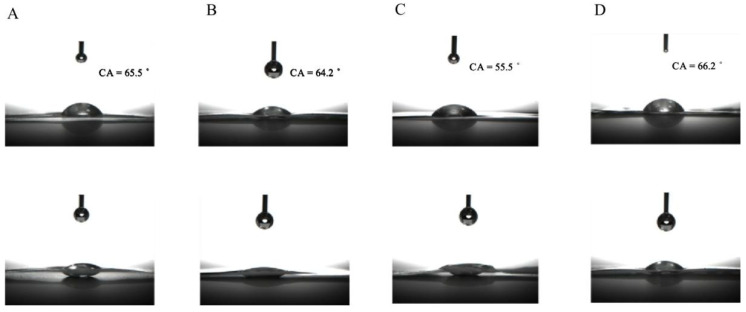
Contact angle of CNC nanocomposite film solutions. CNCs-0 (**A**), CNCs-1 (**B**), CNCs-2 (**C**), and CNCs-3 (**D**) represented the nanocomposite film with 0, 0.01, 0.05 and 0.1% (*w*/*w*) of CNCs incorporation, respectively.

Contact angle of CNCs nanocomposite film suspensions was 65.5°, 64.2°, 55.5°, and 66.2°, respectively (Figure 2). Contact angle of film-forming suspensions on the hydrophobic surface was decreased due to their increased hydrophobicity. The decreased contact angle of CNCs nanocomposite film could be explained by the increased active sites for electrostatic interactions and hydrogen bonds among CS NPs, PS and CNCs. This, in turn, increased the number of hydrophilic sites of CS molecules on the surface. In addition, this result might also be explained as well-dispersed CS NPs with less coagulation over negatively charged CNCs aqueous suspensions [32]. Bahar et al. had also reported that the inclusion of CNCs in polypropylene composite film decreased the contact angle of the film [33]. However, contact angle of CNCs-3 presented higher value than that of CNCs-2 [34]. CNCs are more hydrophilic than the base polymers, the hydrophilicity of CNCs arose due to the exclusion of non-polar components, the insertion of polar sulfate groups, and the exposure of hydroxyl groups from cellulose structure during isolation process [35].

### 2.2. FESEM Observation of CNCs Nanocomposite Film

**Figure 3:**
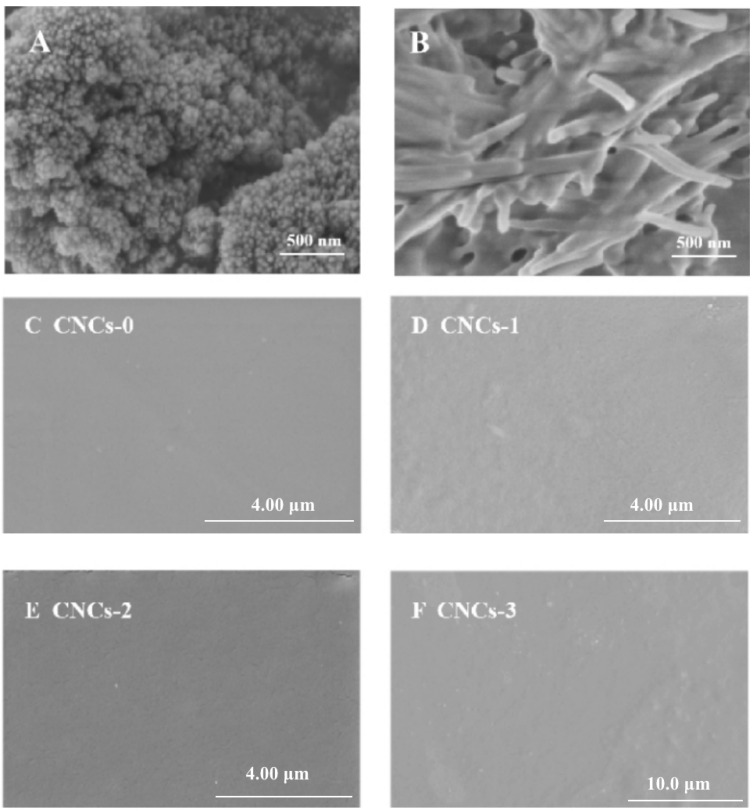
SEM observation of CS NPs (**A**) and CNCs (**B**). Surface morphology of CNCs nanocomposite film. CNCs-0 (**C**), CNCs-1 (**D**), CNCs-2 (**E**), and CNCs-3 (**F**) represented the nanocomposite film with 0, 0.01, 0.05 and 0.1% (*w*/*w*) of CNCs incorporation, respectively. CNCs: cellulose nanocrystals; CS NPs: chitosan nanoparticles.

CS NPs with evenly distributed, though a few agglomerates were observed probably during freeze-drying process (Figure 3A). Individual CNCs had been demonstrated as needle-like shape, consistent with the previous studies (Figure 3B) [25,36,37]. Overall, all CNCs incorporated films (CNCs-1, CNCs-2 and CNCs-3) had similar smoothness and uniform surface compared to the film without CNC incorporation (Figure 3A–D). The rod-like CNCs was evenly distributed in the PS film-forming matrix. The surface of CNCs nanocomposite film became rougher along with elevated CNCs concentrations. Though the successful incorporation of CNC and CS NPs had been demonstrated to the PS matrix based on the interpretations from XRD and contact angle, the amorphous PS entanglement and its higher magnitude in all dimensions compared to CNC and CS NPs avoided the exposure of both nanoparticles in SEM image of surface (Figure 3C–F) and cross sections of composite. Moreover, CNCs-2 nanocomposite film exhibited a darker color, showing consistent observation with the light transmittance result in Figure 1D.

Previous study has reported that the incorporation of 1.5 and 2.5% of CNCs in starch films showed homogeneous and smooth surface [37]. Liu et. al. also studied that the effect of CNCs incorporation on the physicochemical properties of film and suggested that anionic CNCs led to stable and uniform surface morphology due to the attractive or repulsive forces in the matrix [38]. The reasons for the formation of smooth CNCs nanocomposite film can be attributed to: (1) CNCs with low diameter and the high surface charge (strong electrostatic repulsions) might form Pickering emulsion system, resulting in good dispersion properties, and (2) the -OSO_3_– groups at CNCs interacted with -NH^3+^ at CS molecules due to electrostatic attractions.

### 2.3. XRD Analysis of CNCs Nanocomposite Film

**Figure 4:**
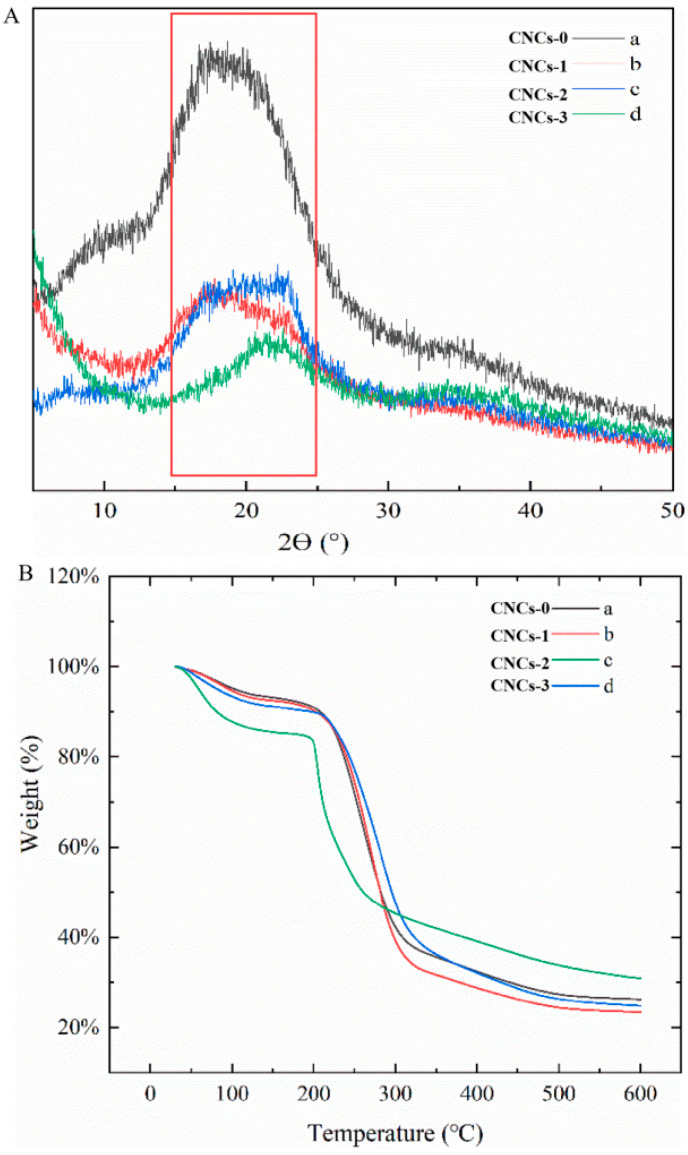
XRD (**A**) and TGA (**B**) analysis of CNCs nanocomposite film. CNCs-0 (a), CNCs-1 (b), CNCs-2 (c), and CNCs-3 (d) represented the nanocomposite film with 0, 0.01, 0.05 and 0.1% (*w*/*w*) of CNCs incorporation, respectively.

XRD spectra of CNCs nanocomposite film are shown in Figure 4A. The diffraction diagram of CS NPs/PS film exhibited two main peaks at 2θ = 11° and 22.78°, which corresponded to [101] and [002] atomic planes of the hydrated crystalline structure of cellulose and amorphous structure of CS, respectively [39,40]. CNCs presented a CrI of 86.2%, indicating that the extraction steps were effective in removing a significant fraction of amorphous structures (hemicelluloses and lignin) during acid hydrolysis [41]. Acid molecules were more susceptible to participate the cleavage of glycoside bond at cellulose amorphous regions than the more well-aligned crystalline domains throughout the diffusion process in aqueous phase [42]. XRD results confirmed that CrI of CNCs film could be decreased with the incorporation of CNCs through hydrogen bonds and electrostatic interactions among CNCs, CS and PS. The peak intensity also increased with elevated cellulose content [43], due to the transcrystallization effect of CNCs [44].

### 2.4. TGA Analysis of CNCs Nanocomposite Film

The first peak at around 60–120 °C was caused by the evaporation of physically adsorbed and strongly hydrogen-bonded water from CS NPs and PS [45]. A significant weight loss in CNCs nanocomposite film was observed around 120–400 °C, because of the depolymerization of CS and CNCs chains through deacetylation and cleavage of glycosidic linkages via dehydration and deamination (Figure 4B). For all films, a major weight loss was found at around 296 °C due to fast volatilization of polymer segments owing to thermal scission of the polymer backbone [46]. The shifted peak values among films were probably because of the incorporation of different CNCs content. It was noted that the demonstrated agglomeration status of CNCs-2 incorporation resulted lower crystallinity and integrity of composite materials, leading to decreased degradation temperature. After thermal decomposition, nanocomposite film without adding CNCs had a higher remaining weight than that of CNCs incorporated film.

### 2.5. Antibacterial Performance of CNCs Nanocomposite Film

**Figure 5:**
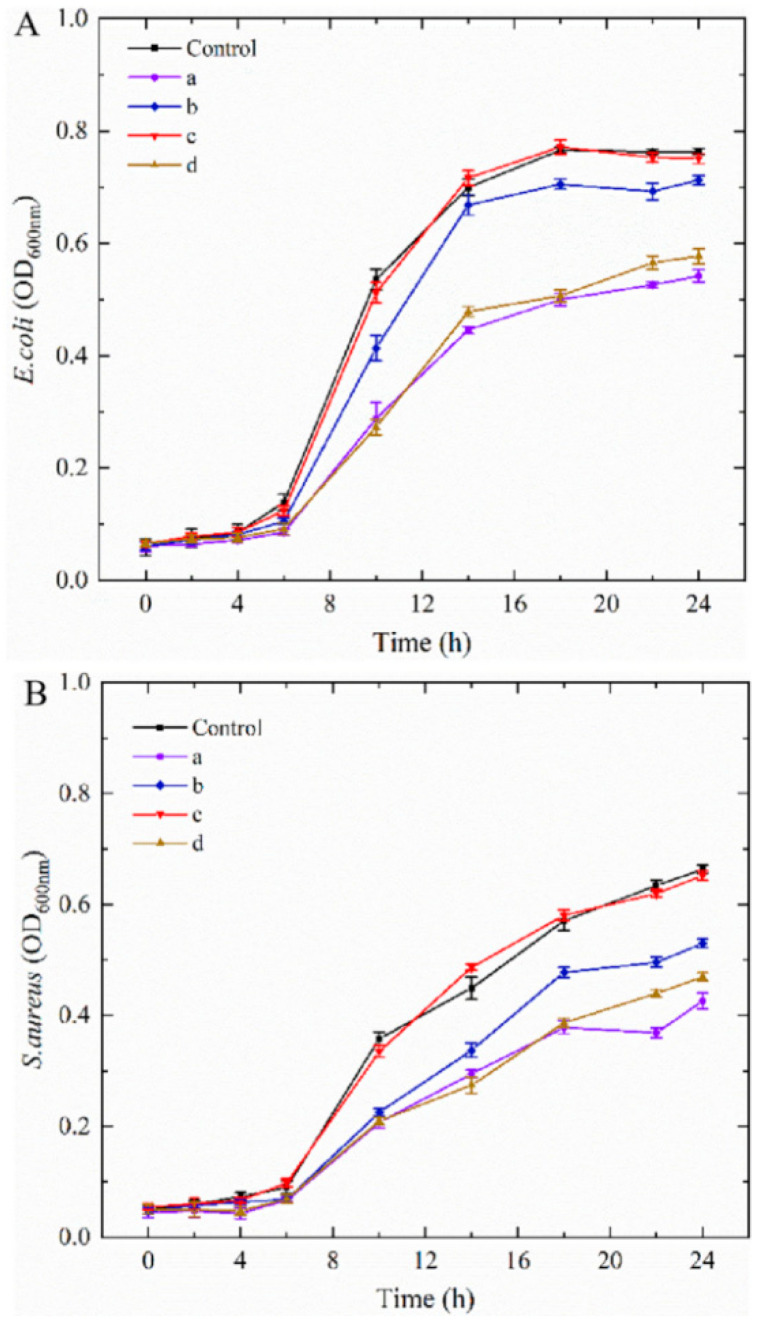
Antibacterial activity of CNCs nanocomposite film against *E. coli* (**A**) and *S. aureus* (**B**). Control: CS NPs; a, b, c, and d represented the nanocomposite film with 0, 0.01, 0.05 and 0.1% (*w*/*w*) of CNCs incorporation, respectively.

The order of overall antibacterial activity against *E. coli* was: controls < CNCs-2 < CNCs-0 < CNCs-3 < CNCs-1 (Figure 5). These results suggested that CNCs-1 nanocomposite film have better antibacterial activity against *E. coli* than others. The poor antibacterial activity of CNCs-2 was due to the aggregation of CNCs, resulting in the agglomeration of CS NPs, showing consistent trends with Figure 1D and Figure 3E. Previous studies had reported that NPs with ~100 nm in diameter had more than three-fold greater arterial uptake capacity compared to the particle in larger size (~275 nm) [47,48]. Therefore, developing dispersible CS NPs with smaller particle sizes is critical to improve their antibacterial properties. Moreover, smaller NPs could easily permeate into cells, leading to the leakage of intracellular substances and interrupt the synthesis of cell membranes and intracellular protein, and consequently kill bacteria strains [49].

The order of overall antibacterial activity against *S. aureus* was: controls ≈ CNCs-2 < CNCs-0 < CNCs-3 ≈ CNCs-1. This result suggested that CNCs-2 has poor antibacterial activity against *S. aureus* as well; meanwhile, CNCs-1 and CNCs-3 showed better antibacterial activities. The following reasons can be attributed to (1) high ZP of CNCs derived from the introduction of -OSO_3_– groups during acid hydrolysis, resulted in large electrostatic repulsion (2) CNCs as enhanced emulsifiers or fillers was added in the CS NPs and PS film-forming matrix for preventing CS NPs from aggregation; thus, enhancing the antibacterial activity [25]. It was noted that the antibacterial activity of CNCs-only film was also reported in our previous study, demonstrating the absence of antibacterial activity from CNCs against both gram-positive and negative microbes [50].

## 3. Materials and Methods

### 3.1. Materials and Reagents

Microcrystalline cellulose (diameter: 100 μm) derived from cotton and PS were purchased from Shanghai Sangon Biotech Co., Ltd. CS with 90% deacetylation degree and 45.25 kDa was from Shanghai Yuanye Biological Technology Co., Ltd. (Shanghai, China). *E. coli*, ATCC 25,922 and *S. aureus* ATCC 25,923 were purchased from ATCC, Rockefeller, MA, USA. The brain heart infusion (BHI), tryptone soybean broth (TSB) and other reagents were purchased from Sinopharm Chemical Reagent Co., Ltd (Shanghai, China). The glass-bottom dishes and centrifuge tubes was obtained from NEST Biotechnology Co., Ltd. (Wuxi, China). All reagents used were analytical grade, deionized water was used in this study.

### 3.2. Preparation of CNCs from MCC

CNCs were isolated from MCC by acid hydrolysis, and the extraction procedures are as described by Baek et al., 2019 [51]. Briefly, aqueous MCC suspension (10 g/100 mL) was blended with a diluted sulfuric acid solution (64 wt%) at 45 °C for 2 h. The recovered material was washed with deionized water until pH reached neutral. The material was then centrifuged (10,000 rpm, 20 min at 20 °C) until the appearance of a colloidal suspension. The supernatant was dialyzed against distilled water to eliminate any residual acids. The extracted CNCs were stored at 4 °C after homogenization. The diameter, polydispersity index (PDI), crystallinity index (CI) and Zeta potential (ZP) of obtained CNCs were 65 nm, 0.21, 86%, and of −43 mV, respectively [41]. For the corresponding analysis, diameter and PDI and ZP were measured using a phase analysis light scattering (DLS) zeta potential analyzer (Zetasizer Nano ZS 90, Malvern, UK) at a 90 ° scattering angle by adjusting initial pH to 5 ± 0.5 with vortex-mixing. Crystallinity index (CI) was calculated from X-ray diffraction (XRD, D8 Advance X, Bruker, Germany) with the Segal method conducted with Cu Ka at 40 kV and 1.54 A.

### 3.3. Preparation of CS NPs Solutions

The 1% of CS solutions (*w*/*v*) were prepared by dissolving CS in aqueous acetic acid (0.1 mol/L). The solution was allowed to stir at 600 rpm, for 24 h followed by filtration through a 0.45 µm membrane to remove the insoluble residue. The stirring rotated at the bottom of the solution using stirrer bar (17 × 50 mm). To prepare CS NPs suspension (0.5% *w*/*v*), 3 mL of 0.25 mg/mL aqueous sodium TPP solution (pH 7.0) were added to 15 mL of 0.25 mg/mL CS solution (pH 4.5) under stirring conditions (600 rpm) for 10 min [19]. The reaction was carried out for 30 min to obtain CS NPs suspensions. Particle size, zeta potential (ZP), and polydispersity index (PDI) were measured to be 71.27 nm, 10.37 and 0.19, respectively [25].

### 3.4. Preparation of Nanocomposite Film with CNCs Incorporated CS NPs in PS Matrix

The 1% of PS was heated at 90 °C for 30 min until complete gelatinization and was allowed to stand at 50 °C to prevent solidification. The CS NPs suspension as film-forming suspensions and gelatinized PS, was added and blended at 600 rpm for 5 min. Different concentrations of CNCs (0, 0.01, 0.05, and 0.1% *w*/*w* labeled as CNCs-0, CNCs-1, CNCs-2, and CNCs-3, dry basis), were added to the above mixture to prepare the nanocomposites film. The mixture solution was stirred at 60 rpm for 30 min after adding glycerol (20% *w*/*w* on a wet basis of PS) [32,52]. Each suspension (170 g) was then cast onto a Teflon coated plate (170 × 170 mm) after degassing process and dried at room temperature for 48 h [50]. Prepared films were conditioned at 25 °C and 50% RH for 2 days prior to all measured parameters.

### 3.5. Physicochemical Analysis of CNCs Nanocomposite Film

Thickness of CNCs nanocomposite film was measured using a hand-held micrometer with a sensitivity of 0.01 mm at five random positions for each sample. Contact angle (°) was determined based on the reported method by Sahraee, Ghanbarzadeh, Milani, & Hamishehkar, 2017 [28]. Contact angle was evaluated to estimate the hydrophilic properties of CNCs-based film suspensions. Each suspension (170 g) was then casted onto a Teflon-coated plate (170 × 170 mm) and dried at ambient temperature for 48 h.

Elongation at break (EAB) and tensile strength (TS) of film were determined using a texture analyzer adhering to the ASTM D882 standard (ASTM, 2001). The initial grip separation and crosshead speed were set to 50 mm and 0.4 mm/s, respectively. Barrier properties of CNCs nanocomposite film against visible light using a spectrophotometer were investigated by measuring the transmission values at a selected wavelength range between 200 and 800 nm. Rectangular pieces were cut out of the samples and were placed perpendicularly in a glass cuvette [28]. A high opacity value means a lower amount of light can pass through CNCs nanocomposite film.

### 3.6. Field Emission Scanning Electron Microscope (FE-SEM)

Microstructure of cross-sections and surface area of CNCs nanocomposite film was investigated by FE-SEM (FEI Quanta 600F, Corvallis, OR, USA). The fractured sample obtained from the mechanical measurements was used for imaging the cross-section morphology. Prepared sample was mounted on aluminum stub with the cross-section oriented up and coated by gold palladium alloy sputter coater (Cressington Scientific Instruments Ltd., Watford, Hertfordshires, UK) to improve the interface conductivity. Digital images were collected at an accelerating voltage of 4.0 kV.

### 3.7. Structural Analysis of CNCs Nanocomposite Film

Fourier transform infrared (FT-IR) spectra of CNCs nanocomposite film were determined using an FT-IR spectrometer. The samples were powdered, mixed with KBr, and pressed into small pellets (99:1, *w*/*w*). The obtained spectra span between wavenumbers ranging from 4000 to 500 cm^−1^ with 100 scans recorded at a 4 cm^−1^ resolution. Thermogravimetric analysis (TGA) was conducted in an automatic analyzer TGA-2000. The 0.5 g sample was heated at a heating rate of 10 °C min^−1^ from 20 to 600 °C under N_2_ protection. The crystalline structures were analyzed by X-ray diffraction (XRD) on a D8-Discover X-ray diffractometer (Bruker, Germany) using Cu Kα radiation at 40 kV and 30 mA in the region of 2θ = 5–50° with a scan rate of 2°·min^−1^.

### 3.8. Determination of Bacterial Growth Rate Using CNCs Nanocomposite Film

Aliquots (100 µL) of *E. coli* and *S. aureus* cell suspensions were respectively added to the 96-well plates (Costar, Bodenheim, Germany). Six film specimens (35 mm^2^) were added into test tubes containing 10 mL of sterilized BHI and TSB culture, respectively, and then inoculated with 100 mL of activated bacterial suspensions (~107 CFU/mL). The absorbance was detected at 660 nm for each well plate at set time intervals [50].

### 3.9. Data Analysis

The obtained data were analyzed by ANOVA using SPSS software. The significant differences were analyzed with Tukey’s test. All measurements were conducted in triplicates, and results ± standard error were reported and considered to be significantly different (*p* < 0.05).

## 4. Conclusions

In this work, CNCs (with varied concentration of 0.01, 0.05, and 0.1%) and CS NPs were successfully incorporated into PS based film-forming matrix for the preparation of the antibacterial nanocomposite film. Developed films presented smooth and uniform surface with compacted cross-section morphology. CNCs incorporation, especially at 0.05%, led to reduction of EAB and light transmittance values of the nanocomposite film. Although no significant enhancement, CNCs addition presented slightly elevation of TS due to the entangled polymeric structure with electrostatic interactions and hydrogen bonds between three major components including CNCs, CS, and PS matrix. The low CNCs incorporation (0.01%) had highest overall antibacterial activity against both *E. coli* and *S. aureus*. It was speculated that CNCs could act as a filler for PS matrix to improve mechanical properties or a Pickering emulsifier for the delivery of CS NPs for enhanced antibacterial properties at low-to-medium incorporation concentration. This study indicated that CNCs incorporated nanocomposite film can be serve as potential candidate for food packaging requiring mechanical strength and elimination of microbial growth. Together with superior enhanced properties as nanomaterial and derived intrinsic properties from biomass, the strong interfacial interactions between spherical CS NPs and rod-shaped CNC will provide superior packaging performance with sustainability, biodegradability and non-toxicity for being potentially scaled-up in low carbon emission of life cycle assessments (LCA).

## Data Availability

All the data generated in the study is reported in the manuscript.

## Sample Availability

Samples of the materials tested are available from the authors.

## Abbreviations

CNC: cellulose nanocrystals; CS: chitosan; PS: potato starch; TPP: triphosphate; NPs: nanoparticles; PDI: polydispersity index; TS: tensile strength; EAB: elogation at break; CrI: crystallinity index; SEM: scanning electron microscopy; XRD: X-ray diffraction; CrI: crystallinity index; TSB: tryptone soybean broth.
